# 

**Published:** 1845-06

**Authors:** 


### INDEX.

PAGE.

A

Anomalies of the Teeth, . . . 63
American Society of Dental Surgeons, Constitution and By-laws of, . . . . . 65
Anatomy, A System of Human, &c. &c., by Erasmus Wilson, M. D., 2d Am. Ed., by Paul Goddard, A. M., M. D. . 71
Address, Opening, by James Taylor, M. D., . . . . 91
Address, Opening, by Solyman Brown, M. D., . . . . 80
Address, before the Mississippi Association of Dental Surgeons, by Dr. John Allen, . 105
Arsenic in Teeth, on the use of, by Hudson S. Burr, M. D., 127
Arsenic in destroying the sensibility of Teeth, on the use of, by Edward Taylor, M. D., . 129
Address, by Jas. Taylor, M. D., 133
Africa, Drawing Teeth in, . 158
Arms, S. C., M. D., Essay on Odontalgia, its causes, &c., 204
Advertisements, Rejection of Quack, . . . . . 234
Alabama, Law regulating the practice of Dentistry in, . 248
Artificial Teeth, Application of, upon the atmospheric or suction principle, by Wm. Harnett, Dentist, . . . . 307
Artificial Incubation, . . . 312
Adhesive Plaster, Prestat's, . 320
American Society of Dental Surgeons, sixth annual meeting of the, . . . . . 322
American Society of Dental Surgeons—Misapprehension, 322

B

Baltimore College of Dental Surgery, . . . . 57—249
Brewster, George G., D. D. S., Obituary, by, . . . . 123
Brewster, Low Fees and Cheap Stock, by, . . . . . 126
Burr, Hudson S., M. D., on the use of Arsenic in teeth, . 127

PAGE.

Blow-pipe and Furnace, Dr. Somerby's, . . . . . 226
Blandin's Human and Comparative Dental Anatomy, . 245
Brockett, L. P., on General Nervous Debility induced by Decayed Teeth, . . . 299
Boston Society of Natural History, . . . . . 312
Budhist, Perpetual Fast of a, 214

C

Coincidence, Singular, . 80
Claims of Medical Science upon the Practitioner of Dental Surgery, Dissertation on the, by Amos Wescott, M. D., . 3
Calculus, Dissertation on, by Wm H. Dwinelle, . . 32
Cook, J. W., M. D., Letter from, . . . . . 49
Congenital Hare-lip, double, by J. M. Sims, M. D. . . 51
College of Dental Surgery, Baltimore, . . . 57—249
College of Dental Surgery, Cincinnati, . . . . 248
China, Progress of Surgery in, 62
Constitution and By-laws of the Am. Soc. of Dental Surgeons, 65
Convention of Dentists in Cincinnati, . . . . . 112
Cyclopedia of Practical Medicine, . . . . . . 159
Cleft Palate and its Treatment, by Dr. S. P. Hullihen, . 166
Case, in which 16 needles, &c. were extracted from the wrist, 231
College, Jefferson Medical, 81—234
Catalepsy, with Convulsions of the right arm, Case of, . 234
Character, value of, . . 236
Copeland's, James, M. D., Dictionary of Practical Medicine, with additions by Chas. A. Lee, M. D., . . . . . 241
Comparative Dental Anatomy, Blandin's Human, . . . 245
Correspondents, To, . . . 80
Calculi, Nasal, . . . . 313

PAGE.

D

Dissertation, &c., by Amos Wescott, M. D., . . . . 3
Dwinelle, W. H., on Salivary Calculus, . . . . . 32
Double Congenital Hare-lip, by J. M. Sims, M. D., . . . 51
Dental Surgery, Baltimore College of, . . . . 57—249
Dental Surg'y, Cincinnati College of, . . . . 248
Dental Surgery, Principles and Practice of, by C. A. Harris, M. D., . . . . . 236
Dentition, Triple, Remarkable Case of, . . . . . 64
Dental Surgeons, Constitution and By-laws of Am. Soc. of, 65
Dentistry, Contributions to Operative and Mechanical, by W. H. Elliott, D. D. S., 83—163
Dental Surgeons, Address delivered before the Mississippi Valley Association of, by Dr. J. Allen, . . . . . . 105
Dentists, Proceedings of a Convention of, . . . . . 112
Dentists, Transactions of the Virginia Society of, . . . 125
Dentists to all Public Hospitals and Dispensaries, . . 152
Dentists, Multiplication of, . 158
Decayed Teeth, Irregular and, 156
Drawing Teeth in Africa, . 158
Dictionary of Practical Medicine, by J. Copeland, 159—241
Dentistry and Physic, by Amos Westcott, M. D., . . 174
Decayed Teeth, Morbid Effects of, by C. O. Cone, D. D. S., 187
Dental Hygiene, . . . . 244
Dental Specimens, Morbid, . 245
Dental Anatomy, Blandin's Human and Comparative, . 245
Dental Exostosis, . . . 246
Dentists in Prussia, Qualifications of, . . . . . 247
Dentists, Virginia Society of Surgeon, . . . . . 247
Dentistry in Alabama, Law regulating the Practice of, . 248
Dewitt's, Rob't, Surgeon, Principles and Practice of Modern Surgery,. . . . . . 72
Dental Surgeons, Am. Soc. of, 73

PAGE.

Dental Surgeons, Mississippi Valley association of, . 78
Decayed Teeth, General Nervous Debility induced by, by L. P. Brockett, M. D. . . 299
Deciduous Teeth, their exchange for tne Permanent Incisors, by Wm. Lintott, Esq. 308
Dosing and Drugging, . . 316
Dentists, Multiplication of, . 319
Dental Mirror, the, and Brooklyn Annual Visitor, by Martin K. Bridges. D. D. S., . 321

E

Epiglottis in the formation of the voice, on the influence of the, 64
Excision of the Teeth, . . 65
Efforts of Nature to form a Pupil at that point of the Eye where it will be most useful, 65
Elliott, W. H., D. D. S., on Operative and Mechanical Dentistry, . . . 83—163
Explanation, . . . . . 160
Essay on Odontalgia, by S. E. Arms, M. D., . . . . 204
Epilepsy cured by the Extraction of a foreign body from the Meatus Auditorius, . 229
Examiner, Medical, . . . 243
Exostosis, Dental, . . . 246
Electro Magnetism, . . . 323

F

Filing the Teeth, Observations on the Utility of, by John Harris, M. D., . . . . 42
Frink, Dr. John N., Obituary of, 162
Fish-hook removed from the Œsophagus, . . . . 228
Forensic Medicine, Principles of, by Wm. A. Guy, M. D., with notes and additions by C. A. Lee, M. D., . . . 242
Fracture, United, Case of, . 323
Forceps, The, Journal of Dental Surgery and the Collateral Arts and Sciences, London, 321

G

Gambini, Dr., on Spontaneous Separation of the Inferior Maxilla, . . . . . 62
Gossip with our Profession, 81—161
Galvanism, Growing Potatoes by, . . . . . . 314

PAGE.

H

Harris, John, M D., on the Utility of Filing the Teeth, . 42
Harris, C. A., M. D., Principles and Practice of Dental Surgery, notice of, by A. Westcott, M. D., . . . . . 236
Hare-lip, Double Congenital, by J. M. Sims, M. D., . 51
Human Anatomy, a System of, by Erasmus Wilson, M. D., 71
Health and Fecundity of the region around Fort Kent, . 157
Hullihen, S. P., Dr., on Cleft Palate, . . . . . 166
Hygiene, Dental, . . . 244
Hare-lip, is the Negro subject to? 314
Hydrocyanic Acid, Case of Poisoning by, . . . . 315

I

Influence of the Epiglottis in the formation of the Voice, 64
Irregular and Decayed Teeth, 156
Insane, Scientific Lectures for the, . . . . . 233
Itinerant Dentists, . . 249
Items, Professional, . . 326

J

Journal, Southern Medical and Surgical, . . . . . 242
Journal, New Orleans Medical, 243
Journal of Medicine, N. York, 243

L

Low Fees and Cheap Stock, by G. G. Brewster, M. D. . 126
Letter from Dr. A. Vancamp, 131
Law regulating the Practice of Dental Surgery in Alabama, 248
Lectures, Medical, . . . . 158
Letter to the Southern Medical and Surgical Journal, . . 230
Liberia Medical School, . 233

M

Medical Science, Claims of, upon the Practitioner of Dental Surgery, by A. Westcott, M. D., . . . . . . 3
Maxillary Bone, Inferior, Spontaneous Separation of the, . 62
Maryland, University of, . 81

PAGE.

Mississippi Valley Association of Dental Surgeons, Address delivered before the, . . 105
Medical Lectures, . . . 158
Multiplication of Dentists, . 158
Morbid Effects of Decayed Teeth, by C. O. Cone, D.D.S. 187
McCabe, James D., D. D. S., on Osseous Union of the Teeth, 224
Medical School, Liberia, . 233
Medical College, Jefferson, . 234
Medicine, A Dictionary of Practical, . . . . . 241
Medicine, Principles of Forensic, . . . . . . 242
Medical and Surgical Journal, Southern, . . . . . 242
Medical Journal, New Orleans, 243
Medical Examiner, . . . . 243
Medicine, N. York Journal of, 243
Morbid Dental Specimens, . 245
Members American Society of Dental Surgeons, their Privileges and Duties, by Amos Westcott, M. D., . . . 251
Maxilla, Inferior, Malignant Tumor of the, . . . 318
Maxilla, Inferior, Fibrous Tumor of the, . . . . 319
Members of the American Society—Misapprehension, . 322

N

Necrosis, Reproduction of Bone in, . . . . . . 317

O

Operative and Mechanical Dental Surgery, by W. H. Elliott, D. D. S., . . 83—163
Opening Address, by James Taylor, M. D., . . . . 91
Obituary, by G. G. Brewster, D. D. S., . . . . . 123
Osseous Union of the Teeth, by James D. McCabe, D. D. S., 224
Ossification of the Pulp of a Tooth, . . . . . . 245
Ohio College of Dental Surgery, First Annual Announcement of, . . . . . 302
Oil Stone, Prepared American, 323

PAGE.

P

Progress of Surgery in China, 62
Principles of Pathology and Practice of Medicine, . . 71
Principles of Modern Surgery, 72
Protrusion of the Under Jaw, Operation for its Correction, by the Syracuse Editor, . 147
Profession, Gossip with our, by the Washington Editor, 81—161
Proceedings of a Convention of Professsonal Dentists, . . 112
Practical Medicine, the Cyclopedia of, . . . . . . 159
Patent Blow-pipe and Furnace, 226
Principles and Practice of Dental Surgery, . . . . . 236
Popular Treatise on the Teeth, 246
Practice of Dentistry in Alabama, Law regulating the, . 248
Pulmonary Consumption, Frequent Spontaneous Cure of, 320
Postage Law, New, . . . 325

Q

Quack Advertisements, rejection of, . . . . . 234
Quackery in High Places, by W. H. Dwinelle, D. D. S., 273
Quack Advertisements, . . 323

S

Surgery in China, Progress of, 62
Spontaneous Separation of a portion of the Lower Jaw, . 62
Society of Dental Surgeons, American, Constitution and By-laws of, . . . . . 65
Society of Dental Surgeons, American, . . . . . 73
Somerby's, Dr., Blow-pipe and Furnace, . . . . . . 226
Surgery, Dental, Principles and Practice of, . . . . . 236
Surgeon Dentists, Virginia Society of, . . . . . 247
Scientific Lectures for the Insane, . . . . . . 233
Subscribers, to, . . . . 80

PAGE.

Sims, J. Marion, M. D., on Double Congenital Hare-lip, 51
Scarification of the Gums, reasons for it, . . . . 311

T

Teeth, Observations on the Utility of Filing, . . . . 42
Teeth, Anomalies of the, . 63
Teeth, Excision of the, . . 65
Taylor, James, M. D., Opening Address by, . . . . 91
Taylor, Ja's, M. D., Address by 133
Taylor, Edward, M. D., . . 129
Teeth, Irregular and Decayed, 156
Teeth, Osseous Union of, . 224
Tooth, Ossification of the Pulp of a, . . . . . . 245
Threat explained, . . 246
Tooth-ache, Lecture on, by E. Saunders, M. R. C. S. . . 279
Teeth, Division of into two classes, translated from the French of Delabarre, . 289
Teeth, Excision of the . . 315
Teeth, Supernumerary, . 325
Teeth, Porcelain. . . . 325

U

Utility of Filing the Teeth. . 42
University of Maryland, . . 80
University of New York, . . 81

V

Vancamp, A. Dr., letter from, 131
Value of Character, . . 236

W

Westcott, Amos, M. D., on the Claims of Medical Science upon the Practitioner of Dental Surgery, . . . . 3
Westcott, on the Protrusion of the Under Jaw, . . 147
Westcott, Amos, on Dentistry and Physic, . . . 174
Westcott, Amos, on the Duties and Privileges of Members of the American Society of Dental Surgeons. . . 251

